# Reports on the Hospitals of the United Kingdom

**Published:** 1912-01-27

**Authors:** Henry Burdett


### REPORTS ON THE

### Hospitals of the United Kingdom.

By SIR HENRY BURDETT, K.C.B., K.C.V.O.

### LXI.—York County Hospital.

When we last inspected this institution some sixteen years ago (see The Hospital, August 31, 1895) we ventured to remind all who are interested in hospitals that the early history of this institution should always be studied by those who wish to realise the dangers in a climate like England of a system of artificial ventilation in a great hospital.

### Avoid Artificial Ventilation.

Since then the plenum system has been tried at great cost in a few British hospitals, but the results should convince all practical people that systems of artificial ventilation in British hospitals are best avoided. We further drew attention to the really abominable accommodation then provided for the nursing staff, and recommended the erection of a nurses' home with every modern convenience. Moreover, our suggestions on this point have borne fruit, and the nursing home which our report produced at the York County Hospital is on the whole satisfactory, though the comforts of the nurses when off duty might be further secured by the addition of more easy-chairs and a small library of readable books.

We further drew attention to the faulty equipment of the hospital, and urged that more attention should be paid to the furniture and fittings, many of which were old, out of date, and required renewal. This important recommendation seems to have been disregarded, the furniture in the wards, and especially the lockers, the flock bolsters, the bad and patchy appearance and poor condition of some of the mattresses, and the absence of modern ward-tables, with sufficient accommodation for dressings and other requisites, especially in the surgical wards, still call for prompt action and reform.

### The Stoving of Mattresses.

An extraordinary practice appears to prevail here with reference to the stoving of mattresses. Instead of the mattresses being unpicked, the hair thoroughly purified and the cases cleaned, the whole mattress is stoved entire and then returned to the wards. The evils of this defective plan are that the contents of the mattresses cannot be adequately purified or renewed, nor can they be rendered as fit as they ought to be for use. The process is wasteful, too, for if the heat to which the mattresses are subjected is as great as it ought to be the ticking soon perishes. We do not know any other hospital of importance where this ancient and inadequate system of stoving still prevails. The sooner it is abolished the sooner will the comfort of the patients, the hygienic condition of the bedding, and the economic interests of the hospital be safeguarded. The best-managed hospitals contract with some firm which removes the mattresses, disinfects their contents, and refills the washed ticks, thus making each mattress practically as good as new. Why has this course not been followed at York? The existing system is indefensible hygienically and economically.

### The Present Condition of the Hospital.

The impression we gained from a thorough inspection of this hospital was that the internal administration requires modernising from top to bottom; there seems to be too little direct touch from the centre with the actual duties of each sister, nurse, and probationer. Indeed, we gathered that the efficiency of the management would be materially increased by the introduction of a controlling hand, backed by up-to-date knowledge; for, although there is evidently no intentional inefficiency, slackness, and the absence of modern methods in several directions, are apparent throughout the hospital. We were astonished to find that the men's accident ward with its defective and out-of-date offices was practically as we found it sixteen years ago.

### The Baths and Lockers.

This ought not to be, and steps should be taken to reconstruct this ward and to supply it with modern sanitary accommodation. Several of the baths in various parts of the hospital should be renewed, and care should be taken to see that they are of adequate size. At present some of them are unsightly and unworthy of the York County Hospital. The same remark applies to the patients' lockers throughout the hospital, which are so ancient as to be probably the worst in England. We noticed that some of the slop-sinks have not worn properly, one supplied by a well-known firm being in such a condition after a few years' wear as to make it essential in our judgment for the reputation of the makers that it should be replaced with a new one by them without cost to the hospital.

The verandas added to most of the wards in 1903 have been of service, but they are far too narrow and ought to be widened so as to enable the beds to be placed at right angles to the main building instead of parallel to it as at present. It is remarkable that any firm of architects erecting hospital verandas so recently as 1903 should not have made them as efficient as possible for hospital purposes. The wooden foul-linen shoots which communicate direct from each of the floors to the laundry in the basement should be removed. A careful inspection of them convinced us that, apart from hygienic reasons, they are at present a serious danger to the safety of the buildings owing to the grave risk from fire which they provide. The whole hospital might be gutted if these shoots were once to get alight, as they readily might do.

We believe steps have been taken to remove some of these risks since the date of our visit.

### A Wonderful Basement.

The basement of these old buildings is as remarkable in its way as was that of the old War Office in Pall Mall, and for the same reasons. Here are placed, in addition to the usual store-rooms, a bakehouse, the kitchens, and a very extensive laundry, where the whole of the washing of the hospital is done.

This laundry has been refitted and modernised, and its extent and arrangements are good. Still there is nothing that can be said in the hygienic or modern hospital sense to justify the maintenance of a great laundry, the bakery, and the kitchens in the basement of any large hospital in the present day. The basement bakery makes all the bread consumed in the institution. We would once more call attention to the rooms of the resident medical officers, which might surely be made more comfortable by the addition of some good furniture and a few other articles, so as to make them as attractive and comfortable as possible.

### Finance.

The office administration is satisfactory, and the work is carefully carried out. We hope that Mr. Neden, the Secretary, will shortly introduce the uniform system of keeping the accounts. This change would be an improvement in many ways, making as it does for economical administration and the accurate comparison of the actual cost at York with that of other hospitals of a similar type.

At the present time the income side of the accounts fails to give sufficient prominence to the contributions of the working classes, which exceed the amount received in annual subscriptions each year. We cannot understand why this is so, or why whilst the artisans contribute through their hospital fund upwards of £1,200 a year, Hospital Sunday in the year 1910 only produced £175.

We commend these two rather striking facts to Archbishop Lang, for the beautiful city of York contains a considerable number of well-to-do residents, whilst the business portion of the community, if we may judge from a careful inspection and inquiry, are not only thrifty but highly prosperous.

We rely upon the Archbishop to give a little active personal service in York and the district in the cause of this hospital, for then surely those who at present starve the hospital revenue will acquit themselves like men by remembering the poor and suffering in better proportion to their means and prosperity than they do at present.

